# A study of *KIR* genes and *HLA-C* in Vogt-Koyanagi-Harada disease in Saudi Arabia

**Published:** 2011-12-29

**Authors:** Atia Sheereen, Ameera Gaafar, Alia Iqneibi, Abdelmoneim Eldali, Khalid F. Tabbara, Chaker Adra, Khaled Al-Hussein

**Affiliations:** 1 Histocompatibility and Immunogenetics Research Unit, Stem Cell Therapy Program; King Faisal Specialist Hospital and Research Centre, Riyadh, Saudi Arabia; 2 Department of Biostatistics, Epidemiology and Scientific Computing, King Faisal Specialist Hospital and Research Centre, Riyadh, Saudi Arabia; 3 Division of Ophthalmology, Department of Surgery, King Faisal Specialist Hospital and Research Centre Riyadh, Saudi Arabia; 4 The Eye Center and the Eye Foundation for Research in Ophthalmology, Riyadh, Saudi Arabia; 5 The Wilmer Ophthalmological Institute of The Johns Hopkins University School of Medicine, Baltimore, MD; 6 Harvard Medical School, Transplantation Research Center, Brigham and Women’s Hospital & Children’s Hospital, Boston, MA

## Abstract

**Purpose:**

Vogt-Koyanagi-Harada (VKH) disease is a serious ocular inflammatory autoimmune insult directed against antigens associated with melanocytes. The repertoire of killer cell immunoglobulin-like receptors (KIRs) is known to play a significant role in the pathogenesis of various autoimmune disorders. Accordingly, we sought to determine the incidence of *KIR* genes and KIR ligand (Human leukocytes antigen [*HLA-C*]) interaction in a cohort of Saudi VKH patients and to compare the findings to normal controls.

**Methods:**

A total of 30 patients with VKH and 125 control subjects were included. PCR using sequence-specific oligonucleotide primers were employed to determine the genotype of the *KIR* genes and *HLA-C* alleles.

**Results:**

The frequency of *KIR2DS3* was significantly higher in the VKH patients than in the control group (p=0.048). Two unique genotypes; VKHN*1 and VKHN*2 were observed in the VKH patients and not in normal controls. In addition, the majority of the VKH patients (82%) in this study carry Bx genotypes that encode 2–5 activating KIR receptors. The genotype Bx5 was found to be positively associated with the VKH patients (p=0.053). Significantly higher homozygosity of *HLA-C2* was observed in the VKH patients than in controls (p=0.005). Furthermore, HLA-C alleles-Cw*14 and Cw*17 were significantly prevalent in the VKH patients (p=0.037 and p=0.0001, respectively), whereas, Cw*15 significantly increased in the control group (p=0.0205). Among potential KIR-HLA interactions, we observed KIR2DL2/2DL3+HLA-C1 to be higher in the control subjects compared with the VKH patients (p=0.018).

**Conclusions:**

Our findings indicated that *KIR2DS3* and *HLA*-class I alleles (-Cw*14 and -Cw*17) may play a role in the pathogenesis of VKH disease. Additionally, the predominance of KIR2DL2/2DL3+HLA-C1 in the controls may imply that this KIR-ligand interaction could possibly play a role in the prevention of VKH disease, or could decrease its severity. These observations may contribute to our understanding of the pathogenesis of VKH and other autoimmune diseases.

## Introduction

Vogt-Koyanagi-Harada (VKH) disease is a multisystem disorder characterized by granulomatous panuvetitis and exudative retinal detachment and is often associated with neurologic and cutaneous manifestations. Ocular inflammation may occur and the onset of VKH disease can be associated with aseptic meningitis, as well as with the subsequent development of vitiligo and hearing changes, as part of the putative cell-mediated autoimmune response affecting melanocytes [1,2]. Several human leukocyte antigens (HLA) have been reported to be associated with VKH disease, including HLA-DR4, HLA-DR53, and HLA-DQ4. Strong association with the HLA-DRB1*0405 subtype has been described in Japanese, Korean, and Saudi populations [3–5], but not in the Mestizo population [6]. We recently reported a significant association of the HLA-DRB1*0405 allele with a Saudi VKH cohort. It is likely that other genetic components of the immune system play a role in conferring risk for VKH disease.

Several studies have shown the influence of killer cell immunoglobulin-like receptors (KIRs), and KIR-ligand pairs, in terms of the susceptibility to and outcome of various autoimmune and infectious diseases, such as AIDS [7,8], hepatitis C virus infection [9], tuberculosis [10], leprosy [11], bird-shot chorioretinopathy [12], idiopathic brochiectasis [13], diabetes mellitus [14,15], Systemic lupus erythromatous [16], Scleroderma [17], Sjogren’s syndrome [18], and ankylosing spondilitis [19].

The polymorphic nature of KIR encodes receptors that inhibit or activate natural killer (NK) cells and certain T-lymphocyte subsets [20–22]. The inhibitory KIR (“iKIRs”) 2DL/3DL recognizes distinct HLA class I molecules and triggers signals to stop NK cell killing. Although the ligands for activating KIRs (“aKIRs”) 2DS/3DS are not well acknowledged, certain aKIRs are predicted to bind to the same HLA-class I ligands in a peptide-dependent manner as their structurally related iKIRs [23]. In addition, the aKIRs interact with the DAP-12 molecules, which modulate granulation and cytokine production in NK cells [24]. Based on the genetic content and the pattern of segregation at the population level, *KIR* haplotypes are divided into two groups, A and B. Group A haplotypes are defined by the presence of seven genes, *KIR2DL1*, *2DL3*, *2DL4*, *3DL1*, *3DL2*, *3DL3*, and *2DS4*, and two pseudo genes. However, group B haplotypes show high genetic diversity and are characterized by the presence of more than one aKIRs. *KIR2DL4*, *3DL2*, *3DL3*, and *3DP1* are ubiquitously present in all individuals and are termed “framework” genes.

The activity of NK cells is controlled by the balance of contra-regulatory signals derived from a wide variety of inhibitory and activating receptors [25].The balance in KIR signaling is provided by the recognition of HLA molecules on the surface of target cells, and some of these ligands are already known. Dimorphism at residue 80 of the α1- helix in HLA-C alleles defines two groups of KIR ligands, HLA-C1, Cw*01, *03,*07,*08,*12,*13,*14, and *16- and -HLA-C2, -Cw*02, *04, *05, *06,*15,*17, and *18, which are specific for KIR2DL2/2DL3/2DS2 and KIR2DL1/2DS1, respectively [26]. Genes encoding *KIR* and *HLA* are located on different chromosomes and vary in both number and type. The independent segregation of *KIR* and *HLA* genes results in variable KIR-HLA combinations in individuals, which might ultimately determine the individual’s immunity and susceptibility to disease [27]. Therefore, the objective of the present study was to investigate the association of *KIR* genes and potential KIR-ligand interactions in patients with VKH disease in a cohort of the Saudi population.

## Methods

### Patients and samples

Genomic DNA samples were collected from 30 patients with VKH disease, as reported previously [5], were stored at −80 °C, and were analyzed for the purpose of the present study. A total of 125 controls were selected from the earlier published data [28], which had a complete set of data for *KIR* and *HLA*.

The VKH patients and controls were Saudi nationals from different parts of the Kingdom of Saudi Arabia. The study was reviewed and approved by the Institutional Review Board at King Faisal Specialist Hospital and Research Centre, Riyadh, Saudi Arabia. Written consent forms were duly signed by all subjects included in the study.

### Genotyping of *KIR* and *HLA*

DNA samples from the VKH patients were typed for 14 *KIR* genes and two pseudo genes using a single stranded polymorphism typing system by Dynal KIR genotyping kit (Invitrogen Corp, Carlsbad, CA). *HLA-C* typing was performed using a high-resolution sequence based typing (SBT) kit (Excellerator HLA kit; Qiagen Inc., Valencia, CA), as previously described [28]. Genotyping tests for all novel genotypes were repeated for the purpose of confirmation.

### Data analysis and statistical methods

The phenotypic frequency of each KIR was calculated as the percentage of positive numbers among all individuals. The statistical software package SAS, version 9.2 (SAS Institute Inc., Cary, NC) was used for data analysis. Differences in the frequencies of *KIR* and *HLA-C* genes between controls and the VKH patients were tested by two-tailed Fisher’s exact probabilities, and p<0.05 was considered statistically significant. Odds Ratios (OD) with 95% Confidence Intervals (CI) were calculated for comparisons showing significant differences between the patients and the control group.

## Results

**Figure 1:**
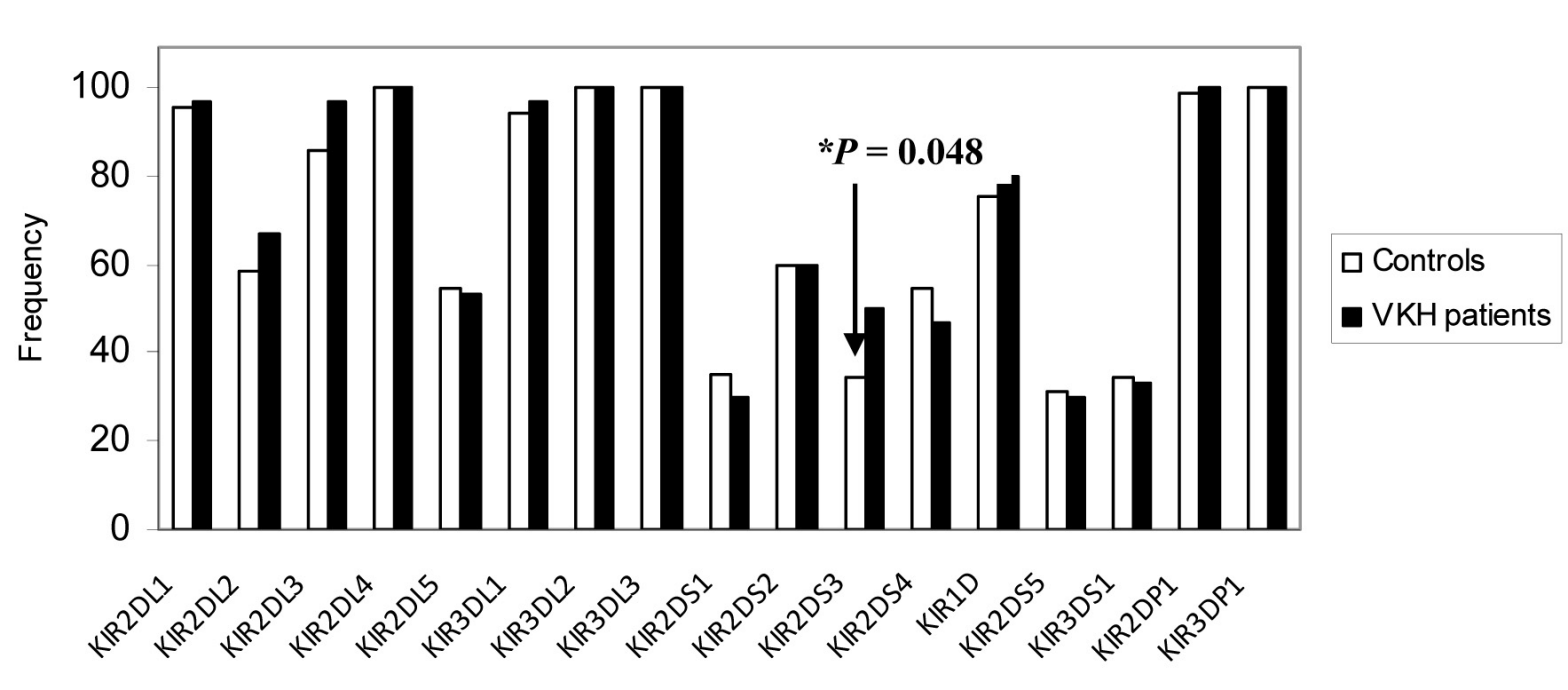
The distribution of killer cell immunoglobulin-like receptor (KIR) gene frequencies in Saudi patients with Vogt-Koyanagi-Harada (VKH) disease and in control subjects. The frequency of each gene is expressed as a percentage and is defined as the number of individuals possessing the genotype (N+), divided by the total number of individuals studied (n). *KIR2DS3 is significantly higher in the VKH patients compared to the controls. The KIR data for the Saudi controls was obtained from our previous publication [28]. The framework genes (*KIR3DL2*, *3DL3*, *2DL4*, and *3DP1*) were invariably present in all individuals.

**Figure 2:**
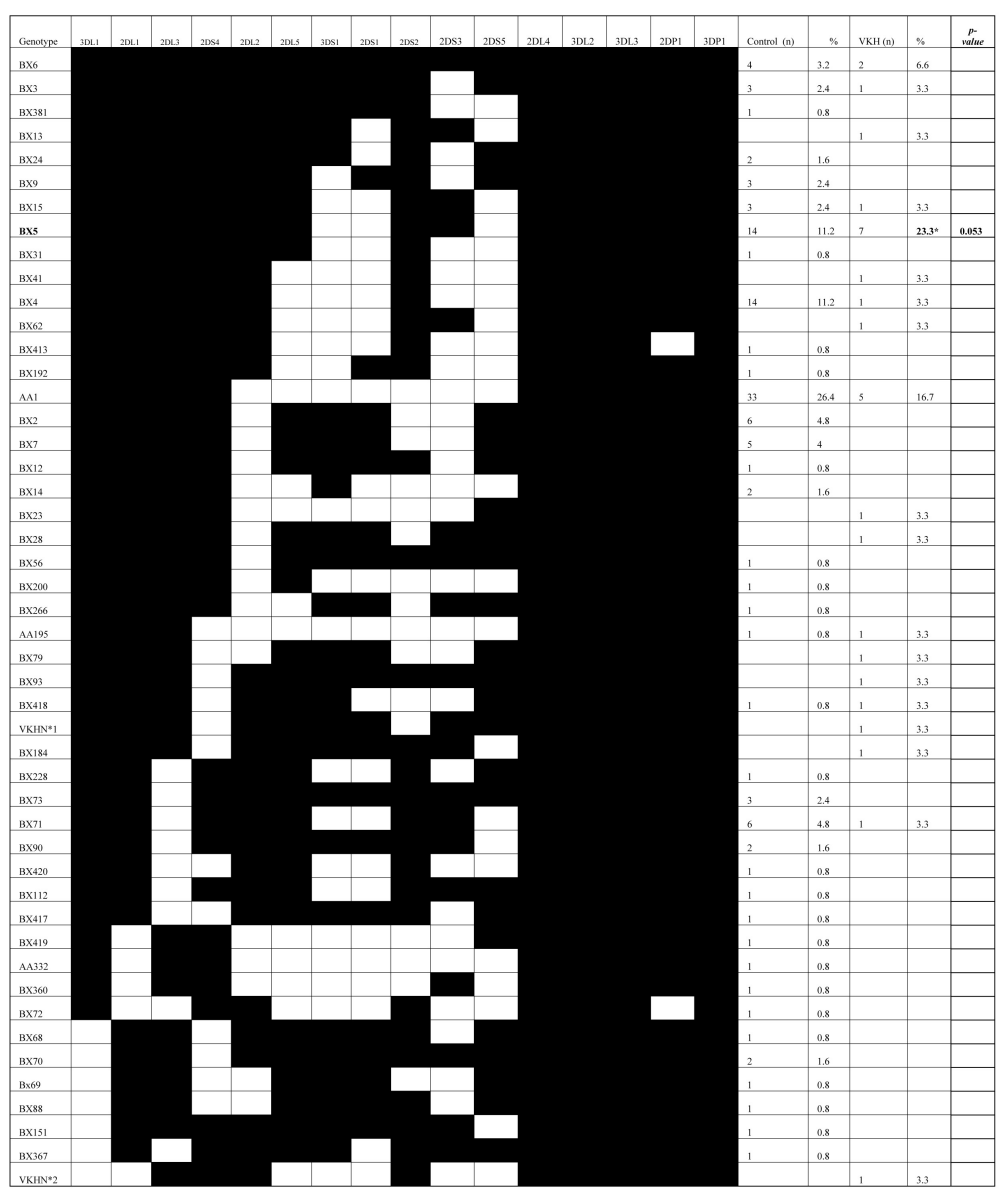
Killer cell immunoglobulin-like receptor (*KIR*) genotypes in Saudi patients with Vogt-Koyanagi-Harada (VKH) disease and in control subjects. Data for the Saudi control individuals was previously published [28]. The frequency of each genotype is expressed as a percentage and is defined as the number of individuals possessing the genotype (+N), divided by the number of individuals studied (n) in each group. VKHN*1 and VKHN*2 are two unique genotypes and are marked with an asterisk. **The frequency of the Bx5 genotype was measured as being significantly higher in the VKH patients compared to the normal controls. (p=0.053; OD=2.41; CI=0.73–7.24).

A total of 30 patients with VKH were studied and compared to 125 Saudi controls who had a complete set of *KIR* and *HLA* data reported earlier [28].The constituents of the control group were not related to the patients or to each other and were all of the same ethnic origin. All of the 16 *KIR* genes were identified in our VKH patients and the controls, and the four framework genes (*KIR2DL4*, *3DL2*, *2DP1*, and *3DL3*) were present in all the subjects tested. The frequencies of *iKIR* and *aKIR* genes in the VKH patients and the controls are outlined in Figure 1. The frequencies of individual *KIR* genes were found to be similar in the controls and the VKH patients, with the exception of *KIR2DS3*, which was significantly higher in the VKH patients compared to the healthy controls (50% in VKH and 34.4% in controls; p=0.048; OD=1.907; CI=0.85–4.26). Furthermore, 19 [19] *KIR* genotypes that differed in terms of their gene content were observed in 30 VKH patients. We observed two unique *KIR* profiles (VKHN*1 and VKHN*2) from two VKH patients that were not found among any of the Saudi control subjects (Figure 2, marked by asterisks). Even though the observed difference did not reach statistical significance, the observation is important as it has not been previously reported elsewhere. Nine [9] genotypes were shared between the VKH patients and the Saudi controls. The frequency of the AA1 genotype was observed to be higher in the controls than in the VKH patients, with a gene frequency of 26.4% and 16.7%, respectively. The ratio of the A:Bx haplotype was found to be 0.33 and 0.20 for the controls and the VKH patients, respectively, (data not shown), indicating the predominance of B haplotype in the VKH patients. Among all the genotypes, only Bx5 reached statistical significance in the VKH patients (p=0.053; OD=2.41; CI=0.73–7.24).

The differences in the frequencies of HLA-C and KIR-HLA-C complex between patients with VKH disease and the control group were statistically significant (Table 1). Significant HLA-C2 homozygosity was measured in the VKH patients (p=0.005; OD=3.23; CI=1.42–7.33). In contrast, the controls were found to have higher HLA-C1 homozygosity than the VKH patients (p=0.029; OD=0.13; CI=0.017–1.009). To explore KIR-HLA class I interactions, the presence of *KIR* genes (*KIR2DL1*, *2DL2*, *2DL3*, *2DS1*, and *2DS2*) and their specific ligands were screened in both the VKH patients and the controls. The frequency of KIR2DL2/3-HLA-C1 interactions was significantly prevalent in the control subjects (p=0.018; OD=0.36; CI=0.16–0.83) compared with the VKH patients (Table 1). Other potential interactions between iKIRs/aKIRs and HLA ligands were found to be similar between the VKH patients and the controls (Table 1). When the frequencies of HLA-C alleles were compared, HLA-Cw *14 (C1 group) and -Cw*17 (C2 group) were observed to be significantly higher in the VKH patients than in the controls (p=0.037; OD=5.31; CI=4.21–6.69 and p<0.010; OD=21.88; CI=4.59–104.29, respectively) (Table 2). However, HLA-Cw*15 (C2 group) was significantly higher in the controls than in the VKH patients (p=0.020; OD=0.308; CI=0.10–0.89) as shown in Table 2.

**Table 1:** Comparison of HLA-class I (-Cw) and KIR - HLA-C complex in a cohort of Saudi patients with VKH disease and control subjects.

	**Controls (n=125)**		**VKH patients (n=30)**			
**HLA-C/KIR-HLA Complex**	**(N+)**	**%F**	**(N+)**	**%F**	**p-value**	**OD 95%(CI)**
HLA-Cw*						
HLA-C1/C1	26	20.8	1	3.3	0.0291	0.13 (0.017–1.009)
HLA-C1/C2	63	50.4	12	40		
HLA-C2/C2	36	28.8	17	56.7	0.0054	3.23 (1.42–7.33)
**KIR-HLA(KIR ligand)**						
KIR2DL2/3-HLA-C1	88	70.4	14	46.7	0.0184	0.367 (0.163–0.830)
KIR2DL1-HLA-C2	97	77.6	28	93.3		
KIR2DS2-HLA-C1	52	41.6	9	30		
KIR2DS1-HLA-C2	32	25.6	8	26.7		

**Table 2:** Frequency of HLA-Cw* alleles in Saudi patients with VKH disease and control subjects.

	**Controls (n=125)**		**VKH patients (n=30)**			
**HLA-C alleles**	**Alleles (n)**	**%**	**Alleles (n)**	**%**	**p value**	**OD 95%(CI)**
cw*01/C1	15	6	2	3.3		
cw*02/C2	8	3.2	2	3.3		
cw*03/C1	10	8	1	1.6		
cw*04/C2	26	10.4	6	10		
cw*05/C1	1	0.4	1	1.6		
cw*06/C2	52	20.8	19	31.6		
cw*07/C1	52	20.8	9	15		
cw*08/C1	10	4	1	1.6		
cw*12/C1	20	8	1	1.6		
cw*14//C1	0	0	2	3.3	0.037	5.3103(4.211–6.696)
cw*15/C2	47	18.8	4	6.7	0.0205	0.3085 (1.06–0.893)
cw*1507/C1	1	0.4	0	0		
cw*16/C1	4	1.6	0	0		
cw*1602/C2	2	0.8	2	3.3		
cw*17/C2	2	0.8	9	15	<0.0001	21.88 (4.59–104.29)
cw*18/C2	0	0	1	1.6		
Total no of alleles (n)	250		60			

## Discussion

KIRs are a relatively recent discovery; while the biologic functions of iKIRs are well described, the function of aKIRs is less clear [29]. Disease association studies have shown that *aKIR* genotypes and KIR-HLA complexes are, in general, associated with a higher risk of autoimmune diseases [27]. We previously reported a significant association of HLA-DRB1*0405 with the VKH patients [5]. Although the clinical manifestations of VKH are well outlined [2], its exact etiology remains to be elucidated. It is believed that T-lymphocyte-mediated autoimmune processes are directed against an, as yet, unidentified antigen or group of antigens associated with melanocytes [1,4,5]. In this study, we found that the VKH patients have a higher frequency of the activating *KIR2DS3* gene compared with healthy controls. Additionally, our results confirm the previous observations made by Levinson et al. [30], who reported a predominance of activating *KIR* genes in Mestizo VKH patients. Although, the observed difference in their study is not statistically significant, it is in keeping with the trend observed in other autoimmune diseases [12]. Interestingly, the preponderance of aKIRs in the VKH patients compared to normal controls suggests that T cell, T cell subsets, and NK cells bearing KIRs might participate in the pathology of the disease. In support of our findings, other studies have demonstrated the association of aKIR genes with a poor prognosis for patients with the Ebola virus infection [31] and other ocular inflammatory disease, like bird shot chorioretinopathy [12]. Moreover, we observed that group B KIR haplotypes predominate in the VKH patients, compared with the controls. However, only the difference in the frequency of the Bx5 genotype reached statistical significance. This might point to a role played by this genotype in the predisposition or the immunopathology of disease. The predominance of the B haplotype in VKH patients was also found to be consistent with other published studies [30]. Two unique genotypic profiles VKHN*1 and VKHN*2 were only detected in the VKH patients and not in the normal Saudi controls and the difference was not found to be statistically significant, as only one individual was measured in each genotype. It is conceivable to postulate that the presence of these two genotypes, alone or in combination with others, has biologic relevance to the disease. Indeed, more investigations are required to prove the previous hypothesis and to identify their exact role in VKH diseases.

It is recognized that the function of KIR is highly dependent on the HLA molecules expressed on the target cells, and both *KIR* and *HLA* show a high degree of polymorphism in the populations. Accordingly, we analyzed the risk conferred by the potential interactions of *KIR* genes with HLA-C in the VKH patients and the control subjects. Only KIR2DL2/3-HLA-C1 was found to be significantly higher in our control population. This was indicative of the inhibitory or protective role played by NK cells. Data from genetic association studies suggest that the signals transduced by aKIRs, upon binding to their putative HLA class I, may serve to overcome HLA class I-dependent inhibition and trigger NK reactivity, leading to an autoimmune condition, as in VKH disease. Although, the precise physiologic ligands for aKIRs are not well defined, KIR tetramer-binding studies imply that activating and inhibitory receptors recognize the same set of HLA class I molecules, but differ in their binding affinities. This allows fine tuning during cellular activation [23]. There is considerable support for the notion that non-HLA molecules may behave as ligands for aKIRs [25]. It is plausible that KIR2DS3 acts by directly recognizing virally encoded proteins, or by recognizing HLA-loaded viral peptide complexes [25]. It is acknowledged that KIR- positive T-cells expressing the adaptor molecule killer cell-activating receptor associated protein (KARAP)/DAP-12, can be stimulated directly with KIR2DS3 to release cytokines and become cytotoxic, without the need for conventional T-cell receptor (TCR) recognition by antigenic peptides [24]. Both KIR and HLA demonstrate a high degree of polymorphism in a given population [20]. In this study, the HLA-Cw*15 allele was more frequently observed in controls, which might point to the role it plays in offering protection from VKH disease. Alternatively, the strong association of the VKH disease with HLA-Cw*14 and -Cw*17* alleles may suggest that the aKIRs, upon binding to their putative HLA-class I ligands, could overcome HLA class I-dependent inhibition and trigger NK cell reactivity, leading to an autoimmune condition such as VKH disease.

Finally, accumulating evidence indicates that aKIRs, and their corresponding specific HLA-C ligands, might contribute to the pathogenesis of VKH disease by modulating NK cells and T cell functions.
